# Care professionals’ perceptions of the use of PainChek^®^ among people living with dementia in Scotland

**DOI:** 10.1186/s12877-025-06784-x

**Published:** 2025-11-29

**Authors:** Isabel Nyangu, Margaret Dunham, Ray Samuriwo, Karen Campbell, Kali Thompson

**Affiliations:** School of Health and Social Care, Edinburgh Napier University, 9 Sighthill Court, Edinburgh, EH11 4BN Scotland, UK

**Keywords:** Dementia care, PainChek®, Pain assessment, Scottish care homes

## Abstract

**Background:**

Moderate to severe dementia is characterised by cognitive decline, communication challenges, and an increased risk of comorbidities, including chronic pain, which is a critical aspect of care. In Scotland, it is estimated that 90,000 people are diagnosed with dementia, 3,000 of whom are aged under 65 years. The Scottish government has been exploring innovative technologies to improve the assessment and management of pain in dementia care.

**Methods:**

We conducted a descriptive online survey with care staff to elicit their perceptions on the use of the digital tool PainChek^®^, for the assessment and management of pain in Scottish care homes as part of a wider evaluation. The study sought to ascertain the outcomes of the digital tool and novel pain assessment strategy, its impact on care, its potential for wider implementation, and the barriers and facilitators to its use. Twenty-eight care home staff who had used PainChek^®^ completed an online survey administered via Microsoft Teams.

**Results:**

PainChek^®^ was perceived by respondents as a user-friendly and effective tool for improving pain assessment and management, especially for non-verbal individuals. Staff reported improved decision-making, more accurate assessments, and a greater capacity to provide person-centred care. Reported facilitators for the adoption of PainChek^®^ included strong management support, comprehensive training, and adequate resources. While respondents generally viewed PainChek^®^ positively, staff were uncertain regarding its related costs.

**Conclusions:**

PainChek^®^ has the potential to improve pain assessment and management in Scottish care homes. Staff highlighted enhanced decision making, more precise pain assessment and its user-friendliness. Its compatibility with national digital strategies and electronic health records could enhance personalised pain management for individuals with dementia across Scotland. We, therefore, recommend continued engagement with staff, providing clear information on how PainChek^®^ can boost efficiency and improve patient-centred care to encourage the continued use of the digital application.

## Background

An increasing number of people in the United Kingdom, including Scotland, are being diagnosed with dementia, which has a negative impact on their health, with a simultaneous rise in the cost of their care. In the UK, 982,000 people have been diagnosedwith dementia, and it is estimated that one in every 14 people aged 65 and above is living with this illness [1]. The percentage of individuals with a formally documented diagnosis varied across the UK. England had the highest rate at 65.4% in 2025, followed by Northern Ireland at 62% by 2022/3, Wales had over half (53.9%) by 2021/2, while in Scotland, it was less than a third (29%) by 2021/22 [1]. The economic impact of dementia was estimated to be £42.5 billion in 2024, and it is projected to more than double to £90 billion by 2040 [1]. In the UK, the estimated costs of social care for people living with dementia in 2024 were £17.2 billion, unpaid care was £21.1 billion, healthcare was £7 billion, and the quality of life economic losses were £2.9 billion [2]. These costs are expected to rise to £40.7 billion, £40 billion, £14 billion and £4 billion in 2040 [2], respectively.

In Scotland, an estimated 90,000 people are diagnosed with dementia, and within this population, approximately 3,000 are aged under 65 years [3]. One-third of people with dementia are in residential care, and they make up 65% of the total care home population [4, 5]. In 2023/24, the Scottish government spent £915.21 million on care services, and £473 million was allocated for individuals aged 65 and over [6].

Dementia remains one of the leading causes of death in Scotland [7]. In 2023, there were 125 deaths per 100,000 people with dementia, more than twice as in 2005 (60 deaths per 100,000) [7]. Most people with dementia died in care homes (63%), compared to just over a fifth (22%) in hospitals, and 14% within a home or non-institutional setting [7]. Deaths attributable to dementia are 1.3 times more likely to occur in the most deprived areas of Scotland [7]. This is highlighted by the fact that Inverclyde and Falkirk had the highest 5-year average mortality rates in Scotland, while Scottish Borders, East Renfrewshire, and North Ayrshire had the lowest in 2023 [7].

The Scottish Government has committed to improving the care of people living with dementia through the Dementia Strategy, which emphasises the importance of person-centred care, enhanced care provider training, the integration of technology into practice, and equitable access to pain resources across the country [3]. While there is significant progress, managing pain among people with dementia is challenging due to barriers in communication, resulting in underreporting and inadequate treatment [8]. It is thought that up to 80% of increased deterioration in cognition has been linked to untreated and continuous pain among people living with dementia [9], and it may also lead to fatalities [10]. As pain management is a critical aspect of dementia care, effective strategies must be tailored for these individuals [11].

Self-report of pain is the gold standard for pain assessment, where people have the physical capacity and language to communicate their pain [12]. In Scotland, pain scales have limited use due to reliance on subjective assessments for those who can communicate, and the lack of routine specialised pain assessment tools tailored for those with cognitive impairment [13]. Pain validated tools, such as the Abbey pain scale and PAINAD scale, can be used among individuals who cannot self-report. Unfortunately, they cannot distinguish between distress and pain and rely on the staff’s interpretation of what the patient is experiencing [14]. PainChek^®^ has been developed as an aid to comprehensive pain assessment, using artificial intelligence (AI) and facial recognition technology to detect and measure pain in individuals who have difficulty reporting. AI-driven algorithms are combined with other data, such as vocalisations, body movements, and physiological changes, to generate a pain score, which is used by health and social care providers to make informed decisions about pain management interventions [15]. Its possible integration with electronic health records and portability are advantages that facilitate its use across various care settings, offering a standardised and novel method of pain assessment [16], enabling immediate access to patient clinical information by healthcare professionals [17] through data sharing, supporting continuity of care, and comprehensive pain management planning [12]. The information collected through PainChek^®^ can be used for audit and quality improvement purposes, allowing for monitoring of pain management practices and outcomes over time [18]. Several Scottish care homes have used PainChek^®^ among people with dementia, and we sought to elicit their perceptions regarding its application.

## Methods

### Aim

The evaluation aimed to assess the utility of PainChek^®^, its impact on care and explore its potential for wider implementation by identifying barriers and facilitators to its use. The objective of the online survey was to explore the perceptions of social care staff working in care homes who accessed PainChek^®^ to assess and manage pain among people with dementia.

### Survey design

To ensure comprehensive data generation, triangulation, and effectively meet the objectives of the evaluation, the evaluation design used a person-centred approach [19] informed by realist principles and appreciative inquiry. This design enabled an inclusive, transparent, and collaborative approach involving a broad range of stakeholders who provided insights, information, and referrals to data sources required by the team to conduct the evaluation. The wider evaluation followed a phased approach, which started with an inception phase during which the terms of reference and communication plan were outlined, and stakeholders were identified. This was followed by a desk review of literature, case studies, and pilot project reports from the Scottish Care Inspectorate, which was conducted to identify best practices for pain assessment and management during dementia care. To explore the perceptions of care staff, survey questions were developed from the results of the desk review.

### Sample and setting

The target population was 50 care home staff experienced in using PainChek^®^. They included care home managers, nurses, and carers. They were sent an email by the Care Inspectorate or the Evaluation team leader, which contained details about the evaluation and an invitation to participate. Before completing the anonymous online Microsoft (MS) survey, there was an agreement statement and an information box with a privacy notice, participant information sheet, and consent checklist, with space after each question for respondents to confirm that they had read and understood. The consent sheet advised on the voluntary nature of participation, and respondents gave consent after they had read the participant information sheet and before completing the survey. A decision to participate or withdraw did not affect their work benefits or professional status, and they could choose not to answer certain questions.

The survey captured information on the perceptions of using PainChek^®^ using a 5-point Likert-type scale (Strongly Disagree, Disagree, Neutral, Agree, Strongly Agree) (see Table 1) and had spaces for respondents to provide any extra information they might want to add. Data were validated for accuracy and completeness before analysis using the Statistical Package for Social Sciences (SPSS) and are presented using descriptive statistics.

**Table 1:** Frequency of responses to survey

Category	Statement	Strongly Disagree	Disagree	Neutral	Agree	Strongly Agree
Perceptions	PainChek^®^ is an effective digital solution for assessing and managing pain.	3.60%	3.60%	10.70%	57.10%	25%
	PainChek^®^ helps to make informed clinical decisions about pain management.	3.60%	3.60%	17.90%	50%	25%
	PainChek^®^ provides accurate pain assessment information that improves the decision-making process.	3.60%	3.60%	25%	57.10%	10.70%
	PainChek^®^ is user-friendly and easy to integrate into everyday work.	3.60%	14.30%	3.60%	46.40%	32.10%
	The layout of PainChek^®^ is efficient and takes minimal effort to navigate.	0%	0%	7.10%	57.10%	35.70%
	PainChek^®^ is a good way of understanding how much pain people who are unable to communicate verbally are experiencing.	3.60%	0%	17.90%	42.90%	35.70%
Benefits	The use of PainChek^®^ has supported me to indirectly improve the quality of care for patients.	3.60%	3.60%	32.10%	39.30%	21.40%
	Using PainChek^®^ has made me feel more confident when assessing and managing my patients’ pain levels.	3.60%	7.10%	28.60%	35.70%	25%
	PainChek^®^ supports a more patient-centred approach to pain management	3.60%	10.70%	21.40%	35.70%	28.60%
Training & Support	I received appropriate training to use PainChek^®^ with my patients.	0%	0%	7.10%	50%	42.90%
	The training provided for PainChek^®^ enabled me to use it confidently	0%	0%	7.40%	51.90%	40.70%
	I can access PainChek^®^ technical support when I need it, if there are any problems.	0%	0%	14.60%	60%	25.40%
Factors for adoption	It is important that management helps and guides staff with the use of PainChek^®^ in services to make it successful	0%	0%	14.30%	46.40%	39.30%
	Training and resources are needed for the successful use of PainChek^®^ in a service.	0%	0%	10.70%	46.40%	42.90%
Cost-effectiveness	Resources required for the use of PainChek^®^ (i.e. training, time, technology) are good value for money.	0%	3.70%	51.90%	37%	7.40%
	The benefits of PainChek^®^ outweigh the cost of using it in a care setting.	3.70%	0%	55.60%	33.30%	7.40%
	The use of PainChek^®^ could help to reduce the chance of patients’ pain going unnoticed.	3.70%	0%	18.50%	51.90%	25.90%
	The use of PainChek^®^ could help to reduce the amount of pain medication needing to be administered	3.70%	0%	18.50%	59.30%	18.50%
Satisfaction	I have noticed the benefits of using PainChek^®^ in my day-to-day work	3.60%	10.70%	21.40%	46.40%	17.90%
	I am happy to continue to use PainChek^®^ in my day-to-day work	0%	14.30%	14.30%	46.40%	25%
	I think PainChek^®^ is helpful in supporting me in assessing and managing my patients’ pain	3.60%	7.10%	14.30%	46.40%	28.60%
	There are some issues/problems with PainChek^®^ that can affect patient care	7.10%	28.60%	32.10%	17.90%	14.30%

### Ethical considerations

To ensure the protection of the respondents, ethical approval of the wider evaluation, including this survey, was obtained from Edinburgh Napier University’s School of Health and Social Care (SHSC) Integrity Committee (SHSC3742809). The well-being and rights of respondents were prioritised by adhering to the Declaration of Helsinki [20]. Respondents provided informed consent after reading the privacy notice, participant information sheet, and consent checklist and before completing the survey. All local and national requirements for confidentiality were adhered to, in full compliance with the General Data Protection Regulation (GDPR) [21]. All research data were anonymised and stored in a password-protected secure online university folder. Alphanumeric identifiers were used to pseudonymise respondents, and these were stored separately from the digital files to ensure the anonymity of all data and compliance with the University’s Data Management Policy.

## Results

There was a 56% (*n* = 28) response rate to the survey. In almost every case, the most popular response was participants agreeing with the statements put forward in the survey about PainChek^®^. (See Table 1)

### Staff perceptions on PainChek^®^

**Fig. 1:**
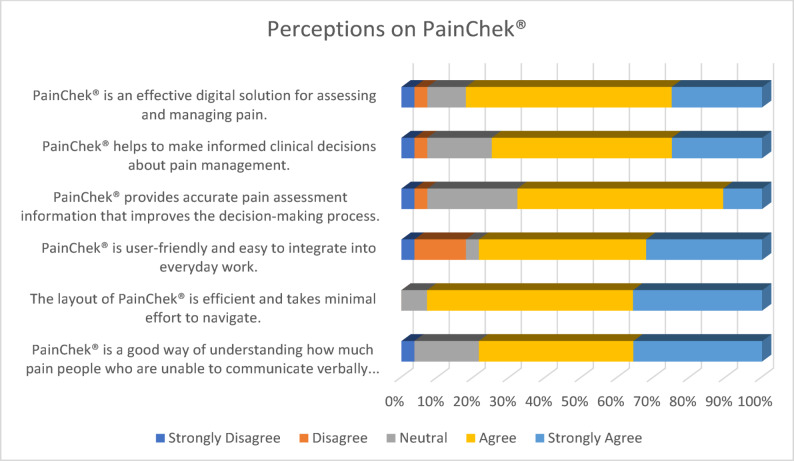
Perceptions on PainChek^®^

Staff were asked about their perceptions of PainChek^®^ to elicit their overall understanding of the tool as a support to assess pain in their patients. Most respondents (82.1%, 16 Agree, 7 Strongly Agree) felt that PainChek^®^ is an effective digital solution for assessing and managing pain. Three-quarters (75%, 14 Agree, 7 Strongly Agree) felt that it helped them to make informed decisions about pain management. Just over two-thirds (67.8%, 16 Agree, 3 Strongly Agree) felt that it provided an accurate assessment of information, improving their decision-making. Almost four-fifths (78%, 13 Agree, 9 Strongly Agree) felt that it is user-friendly and easy to integrate into everyday work, and almost all (92.8%, 16 Agree, 10 Strongly Agree) respondents felt that the layout of PainChek^®^ is efficient and takes minimal effort to navigate. About four-fifths (78.6%, 12 Agree, 10 Strongly Agree) perceived that it is a good way to understand how much pain people who are unable to communicate verbally experience. This suggests that staff who have used PainChek^®^ perceive it positively as a tool for the effective assessment and management of pain in those whom they care for. (See Fig. 1)

### Benefits of using PainChek^®^

**Fig. 2:**
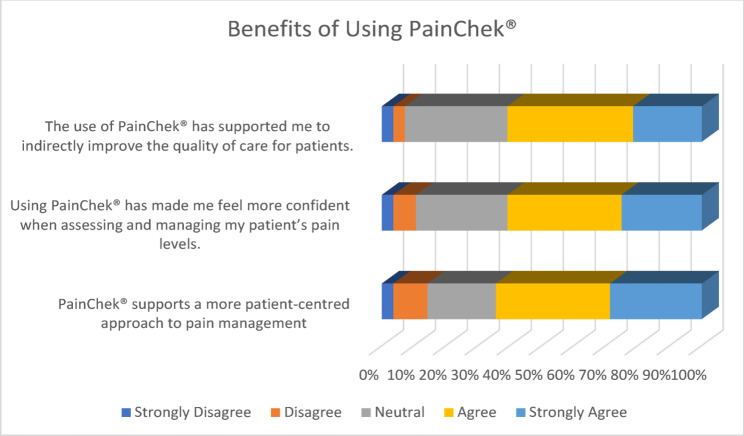
Benefits of using PainChek^®^

Over half of the respondents (60.7%, 11 Agree, 6 Strongly Agree) felt that PainChek^®^ supported them to indirectly improve the quality of care for their patients. Similarly, 60.7% (10 Agree, 7 Strongly Agree) felt more confident when assessing and managing their patients’ pain levels through using PainChek^®^. Moreover, about two-thirds (64.3%, 10 Agree, 8 Strongly Agree) felt that it supports a more patient-centred approach to pain management. Overall, responses would suggest that staff perceived PainChek^®^ to be beneficial in supporting them to assess and manage pain in their patients. (See Fig. 2)

### Training and support before using PainChek^®^

**Fig. 3:**
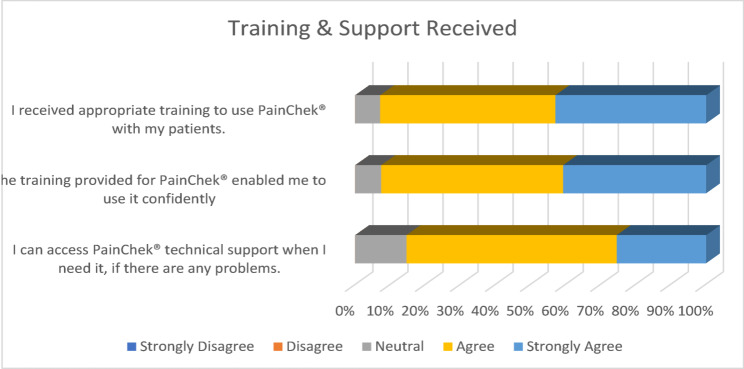
Training and Support Received

Most respondents (92.9%, 14 Agree, 11 Strongly Agree) suggested that they received appropriate training to use PainChek^®^. Similar results were found about their level of confidence, with 92.6% (14 Agree, 11 Strongly Agree) reporting that the training provided for PainChek^®^ enabled them to use it with confidence. Slightly fewer (85.4%, 17 Agree, 7 Strongly Agree) respondents felt that they could access PainChek^®^ technical support when they needed it, if problems arose. None of the respondents (0%) disagreed with any of the statements. (See Fig. 3)

### Factors for the adoption of PainChek^®^

**Fig. 4:**
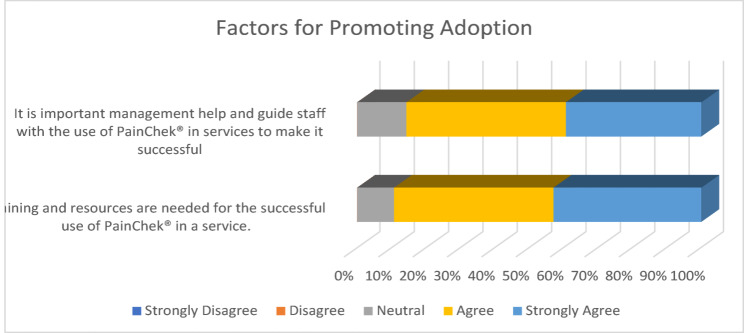
Factors for promoting adoption

When asked about factors that would increase the likelihood of staff continuing to use PainChek^®^ within their workplace, responses ranged from ‘Neutral’ to ‘Strongly Agree’, with no one disagreeing with any statement. Most respondents (85.7%, 13 Agree, 11 Strongly Agree) felt management must help and guide staff with the use of PainChek^®^ in services to make it successful. Some respondents (89.3%, 13 Agree, 12 Strongly Agree) felt that training and resources are needed for the successful use of PainChek^®^ in a service. Again, no respondents (0%) disagreed with any of the statements regarding factors for adoption, suggesting that staff perceived technical and management support, training and resources to be essential for the successful implementation and use of PainChek^®^ in care services. (See Fig. 4)

### Cost-Effectiveness of using PainChek^®^

**Fig. 5:**
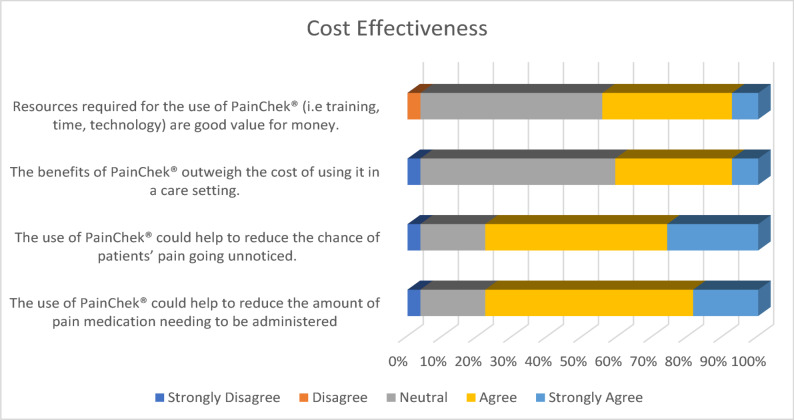
Cost-effectiveness of PainChek^®^

Overall, respondents seemed to be unsure as to whether PainChek^®^ was cost-effective on statements related to cost directly, with over half responding neutral. This may be indicative of staff not being privy to the costs associated with PainChek^®^ and other elements of care, such as medication, hospitalisation, and other support tools, which would make it difficult to be able to perceive how cost-effective PainChek^®^ would be in their care setting. Less than half of respondents (44%, 10 Agree, 2 Strongly Agree) felt that the resources required for the use of PainChek^®^ were good value for money, and the majority (51.9%, 14 Neutral) remained unsure. Similar results were found when asked if the benefits of PainChek^®^ outweighed the costs, with over half (55.6%, 15 Neutral) remaining neutral and two-fifths (40.7%, 9 Agree, 2 Strongly Agree) agreeing with the statements. Despite this, over three-quarters of respondents (77.8%, 14 Agree, 7 Strongly Agree) felt that the use of PainChek^®^ could help to reduce the chances of a patient’s pain going unnoticed. Similar results were reported about the use of PainChek^®^ to help reduce the amount of pain relief medication needing to be administered, with 77.8% (16 Agree, 5 Strongly Agree) agreeing. With improved identification of pain and reduction in pain relief medication, there could be a potential reduction in costs with the use of PainChek^®^. (See Fig. 5)

### Satisfaction with PainChek^®^

**Fig. 6:**
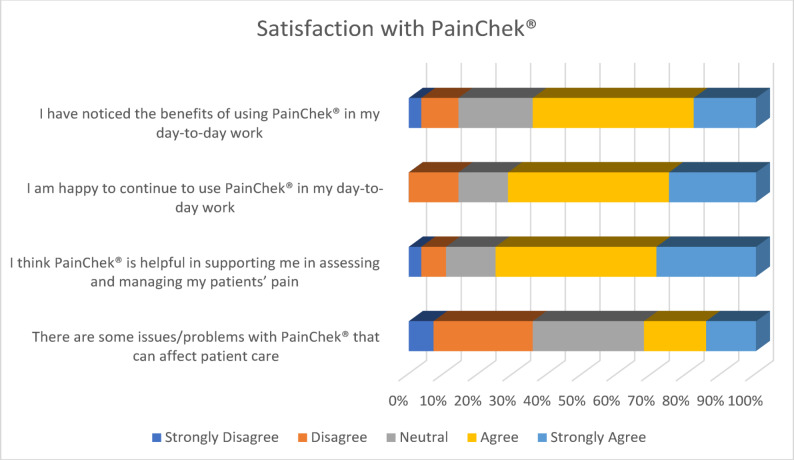
Satisfaction with using PainChek^®^

Over half (64.3%, 13 Agree, 5 Strongly Agree) of respondents agreed that they noticed the benefits of using PainChek^®^ in their day-to-day work. Just under three-quarters of respondents (71.4%, 13 Agree, 7 Strongly Agree) felt that they would be happy to continue to use PainChek^®^ within their day-to-day work. Three-quarters of respondents (75%, 13 Agree, 8 Strongly Agree) felt that PainChek^®^ supported them to assess and manage their patients’ pain. In terms of issues/problems with PainChek^®^ that can affect patient care, over a third of staff (35.7%, 2 Strongly Disagree, 8 Disagree) felt as though there were none. However, almost a similar number of respondents felt neutral (32.1%, 9 Neutral) and just under two-thirds perceived there to be some issues/problems (32.2%, 5 Agree, 4 Strongly Agree). (See Fig. 6)

## Discussion

PainChek^®^ aligns with national strategies in Scotland for improved pain assessment and management in dementia, addressing clinical and ethical concerns. Its possible integration with electronic health records and portability are advantages that facilitate its use across various care settings, offering a standardised and novel method of pain assessment [16]. Staff satisfaction is crucial for the implementation of a new tool in any workplace, and a lot can be deduced regarding the success of integration based on staff response [22]. The feedback from a small sample of health and care professionals in selected care homes in Scotland indicates the utility of PainChek^®^ in assessing and managing pain among individuals with moderate to severe dementia.

In previous studies, PainChek^®^ reportedly reduced the subjectivity of traditional pain assessments [9], improved accuracy in detecting pain, especially in non-verbal individuals [12] and allowed for timely interventions and more standardised measures of pain detection [23]. In this evaluation, staff perceptions of PainChek^®^ were largely positive, with the majority responding that they perceived it to be an effective digital solution for assessing and managing patient pain. The staff who responded perceived it as helpful in making informed decisions about pain management, facilitating accurate assessments, improving their decision-making processes and providing a valuable solution for understanding pain in patients who are unable to communicate verbally. The perceived benefits of using PainChek^®^ were also highly rated, with staff who took part in the survey agreeing that it supported a more person-centred approach to pain management and improved the quality of care. These findings are supported by previous studies, which reported that the provision of patient-centred care encourages autonomy, increases patient satisfaction [16] and promotes equity in healthcare delivery across various settings, such as care homes and hospitals [9]. In addition, using PainChek^®^ allows for regular assessments and provides more responsive and individualised care due to its portability and ease of use [24].

Most staff who took part expressed a willingness to continue using PainChek^®^ as it increased their confidence in assessing and managing patient pain levels in their daily work. Staff acknowledged that PainChek^®^ could reduce the chances of pain going unnoticed and decrease the need for pain relief. This finding is in line with a previous study, which also reported people’s willingness and enthusiasm to use technological devices such as PainChek^®^ when the benefits and ease of use outweigh negative perceptions [25]. However, in this evaluation, while staff considered PainChek^®^ to be user-friendly and have an efficient layout that required minimal effort to navigate, the statement suggesting that PainChek^®^ is easy to integrate into everyday work yielded the highest disagreement rate. This may be due to the busy nature of care settings, which makes it difficult to incorporate new tools and technologies such as PainChek^®^ into an already strained workflow.

Feedback from staff emphasised factors for successful adoption as support from management, training, and resources. Staff agreed that these elements were essential for the effective implementation and use of PainChek^®^. The training and support provided for PainChek^®^ were exceptionally well-received, with no staff members expressing dissatisfaction. The training was deemed appropriate, sufficient, and effective, enabling staff to use the tool confidently, and technical support was considered readily accessible. This finding is supported by a previous study, which alludes to a new pain assessment strategy requiring integration into existing care workflows, as well as training and education, to build confidence in the effectiveness and ease of use of the tool [16]. Fortunately, the training on the use of PainChek^®^ is provided for free in-person and online.

In summary, the staff feedback from the small evaluation sample was largely positive, confirming the tool’s utility, its role in improving decision-making accuracy for non-verbal individuals, boosting staff confidence, and promoting a person-centred approach. The training and support were well-received, fostering willingness among care professionals to continue its use. However, sufficient data were not available to establish the cost-effectiveness of PainChek^®^ in this evaluation. A previous study reported that there are financial implications to the adoption of PainChek^®^, such as costs related to hardware and software [24] and these will have to be considered if implementation is to be successful in Scotland. Unfortunately, the limited response rate (*n* = 28) to the staff survey across care homes where PainChek^®^ has been used makes it difficult to generalise the results of this survey. It is possible that those who were reluctant to use PainChek^®^ or did not have a positive experience using it did not feel comfortable disagreeing with statements in the survey.

## Conclusions

PainChek^®^ has the potential to contribute to pain assessment and its effective management in Scottish care homes. Those staff we contacted had positive perceptions of PainChek^®^. The staff who engaged with the survey noted its user-friendliness, its support for informed decision-making, and the effectiveness of the training and support provided. Furthermore, PainChek^®^ aligns with national digital strategies, may contribute to digital integration in care and shows potential for electronic health record integration and data-driven quality improvement. Critical factors for adoption and implementation include the importance of management and technical support, training and resources. Continued commitment from health and social care providers, policymakers, and researchers will be crucial in overcoming challenges and ensuring equitable access to advanced pain management solutions for people with dementia across Scotland. By leveraging technology alongside traditional care approaches, Scotland can enhance the quality of life for individuals with dementia, providing more personalised and effective pain management that is responsive to their unique needs.

For the wider evaluation, we recommend continued engagement with staff involved in the implementation of PainChek^®^ through the maintenance of open communication channels for staff feedback to address any concerns or suggestions. Staff should be provided with clear information about how PainChek^®^’s data can lead to more efficient resource allocation and reduced overall healthcare costs to address uncertainty and foster more positive perceptions. This may contribute to the adoption of PainChek^®^ by care professionals.

## Data Availability

The data generated and analysed during the survey are not publicly available as they are kept in a password-protected secure university folder, but are available from the corresponding author upon reasonable request.

## Abbreviations

AI: Artificial Intelligence
GDPR: General Data Protection Regulation
MS: Microsoft
SHSC: School of Health and Social Care
SPSS: Statistical Package for Social Sciences
UK: United Kingdom
